# Research and Implementation Lessons Learned From a Youth-Targeted Digital Health Randomized Controlled Trial (the ARMADILLO Study)

**DOI:** 10.2196/13005

**Published:** 2019-09-27

**Authors:** Lianne Gonsalves, Winnie Wangari Njeri, Megan Schroeder, Jefferson Mwaisaka, Peter Gichangi

**Affiliations:** 1 Department of Reproductive Health and Research World Health Organization Geneva Switzerland; 2 Swiss Tropical and Public Health Institute Basel Switzerland; 3 University of Basel Basel Switzerland; 4 International Centre for Reproductive Health-Kenya Mombasa Kenya; 5 Ona Nairobi Kenya; 6 Department of Population, Family & Reproductive Health University of Ghana Accra Ghana; 7 Department of Human Anatomy University of Nairobi Nairobi Kenya; 8 University of Ghent Ghent Belgium

**Keywords:** adolescent health, sexual and reproductive health, health communication, mHealth, Kenya, intervention research

## Abstract

**Background:**

Evidence is lacking on the efficacy of sexual and reproductive health (SRH) communication interventions for youth (aged 15-24 years), especially from low- and middle-income countries. Therefore, the World Health Organization initiated the Adolescent/Youth Reproductive Mobile Access and Delivery Initiative for Love and Life Outcomes (ARMADILLO) program, a free, menu-based, on-demand text message (SMS, short message service) platform providing validated SRH content developed in collaboration with young people. A randomized controlled trial (RCT) assessing the effect of the ARMADILLO intervention on SRH-related outcomes was implemented in Kwale County, Kenya.

**Objective:**

This paper describes the implementation challenges related to the RCT, observed during enrollment and the intervention period, and their implications for digital health researchers and program implementers.

**Methods:**

This was an open, three-armed RCT. Following completion of a baseline survey, participants were randomized into the ARMADILLO intervention (arm 1), a once-a-week contact SMS text message (arm 2), or usual care (arm 3, no intervention). The intervention period lasted seven weeks, after which participants completed an endline survey.

**Results:**

Two study team decisions had significant implications for the success of the trial’s enrollment and intervention implementation: a hands-off participant recruitment process and a design flaw in an initial language selection menu. As a result, three weeks after recruitment began, 660 participants had been randomized; however, 107 (53%) participants in arm 1 and 136 (62%) in arm 2 were “stuck” at the language menu. The research team called 231 of these nonengaging participants and successfully reached 136 to learn reasons for nonengagement. Thirty-two phone numbers were found to be either not linked to our participants (a wrong number) or not in their primary possession (a shared phone). Among eligible participants, 30 participants indicated that they had assumed the introductory message was a scam or spam. Twenty-seven participants were confused by some aspect of the system. Eleven were apathetic about engaging. Twenty-four nonengagers experienced some sort of technical issue. All participants eventually started their seven-week study period.

**Conclusions:**

The ARMADILLO study’s implementation challenges provide several lessons related to both researching and implementing client-side digital health interventions, including (1) have meticulous phone data collection protocols to reduce wrong numbers, (2) train participants on the digital intervention in efficacy assessments, and (3) recognize that client-side digital health interventions have analog discontinuation challenges. Implementation lessons were (1) determine whether an intervention requires phone ownership or phone access, (2) digital health campaigns need to establish a credible presence in a busy digital space, and (3) interest in a service can be sporadic or fleeting.

**Clinical Trial:**

International Standard Randomized Controlled Trial Number (ISRCTN): 85156148; http://www.isrctn. com/ISRCTN85156148

## Introduction

In the past 10 years, the digital health field—or the use of digital, wireless, and mobile technologies for health [1]—has exploded in size and scope of interventions. Digital health solutions have been promoted with enthusiasm due to the technology’s rapid and widespread proliferation, and the potential of digital health to improve access to health information and services, especially in resource-poor settings [2]. Today, the World Health Organization’s Classification of Digital Health Interventions describes a spectrum of solutions for supporting health care providers, health system and resource managers, health data services, and clients of the health system [3].

Young people (individuals between 15 and 24 years) are an especially promising population to reach with digital health interventions. They often face special vulnerabilities, especially related to sexual and reproductive health (SRH). In developing regions, an estimated 33 million women aged 15 to 24 years have an unmet need for contraception [4]; 16 million girls aged 15 to 19 years give birth each year and 3.9 million girls aged 15 to 19 years undergo unsafe abortions [5]. In Kenya, young people between the ages of 15 and 24 years constitute one-fifth of the total population [6]. The most recent Kenya Demographic and Health Survey, found that 37% of young women aged 15 to 19 years and 49% aged 20 to 24 years who are currently married, and 49% of young women aged 15 to 19 years and 64% aged 20 to 24 years who are sexually active but not married, are currently using any form of modern contraception [7]. Even among currently married women, those aged 15 to 24 years still have an unmet need for family planning that is higher than the national estimate of unmet need among all women of reproductive age (15-49 years) [7].

Despite demonstrated SRH needs in Kenya, and around the world, young people have traditionally faced a wide variety of financial, cultural, social, and legal obstacles to obtaining SRH services [8,9]. They are also, however, voracious adopters and innovators when it comes to mobile phone technology [10]. Recent years have seen an explosive proliferation of mobile phone ownership, thus closing ownership gaps across education and wealth levels [11-13]. Kenya leads East Africa in mobile phone infrastructure and innovation (for example, with higher-than-average coverage in rural areas, and long-time use of mobile money programs driving increases in mobile phone access) [13]. As such, youth-targeted digital health solutions appear to be a logical intervention for privately disseminating needed information to a population with special SRH-related vulnerabilities.

Unfortunately, evidence on the efficacy of client-targeted SRH communication interventions for young people, especially in low- and middle-income countries, is severely lacking [14]. Therefore, in 2014, the World Health Organization’s Department of Reproductive Health and Research initiated the Adolescent/Youth Reproductive Mobile Access and Delivery Initiative for Love and Life Outcomes (ARMADILLO) study, joined by the International Centre for Reproductive Health–Kenya, and Kenya-based technology partner Ona. An additional research partner also implemented the ARMADILLO study in Peru.

The ARMADILLO study was envisioned as a proof-of-concept intervention study. The intervention was designed as a free, automated, menu-based and on-demand text messaging (short message service; SMS) platform that would provide validated information across a variety of youth-identified SRH domains. The study itself was implemented in two stages. A formative stage 1 identified relevant SRH domains, and then developed and tested the SMS text messaging content and intervention appeal among youth aged 15 to 24 years with qualitative methods [15]. For the stage 2 efficacy assessment [16], we opted to conduct a randomized controlled trial (RCT), long considered the gold standard in health research study design, so that the ARMADILLO study might address repeated global calls for rigorous evidence that digital interventions can (either directly or as secondary outcomes) positively impact health outcomes [14,17-19].

It is established that conducting RCTs on digital health interventions can be challenging due to the study’s rigid design, as well as the cost and time often required [20]. However, the purpose of this paper is to describe some additional implementation challenges that arose during the ARMADILLO RCT in Kenya. These issues, which arose during the period of enrollment and early during the intervention period, have implications for both digital health researchers and programmers attempting similar, client-side health communication interventions, especially with young people.

## Methods

### Overview

The full procedures for the ARMADILLO trial (registration number: ISRCTN85156148) are described in full elsewhere [16]; briefly, this was an open, three-armed RCT conducted in a peri-urban area in Kwale County, Kenya. The RCT sought to determine whether the provision of on-demand SRH information via text message (arm 1) would result in significant improvement over several SRH knowledge, attitudinal, and behavioral outcomes as compared with periodic messages encouraging self-learning (arm 2) or usual care (no intervention, arm 3). The primary outcome measured change in an index of myths and misconceptions related to contraception. Secondary outcomes measured change in knowledge, attitudes, and behavior for key SRH outcomes (eg, knowledge of HIV/AIDS and its transmission, attitudes around violence against women, engagement in sexual activity).

Following recruitment and consent, participants completed a baseline survey capturing sociodemographic information and primary and secondary outcome measures. Participants were then randomized into a 1:1:1 ratio using a computer-based randomization tool (developed using Node.js and docker). The intervention period lasted seven weeks, at which point data collectors visited participants to administer an endline survey of SRH outcomes. After completing an additional eight-week period, during which no participants received any intervention, participants completed a final, follow-up assessment of SRH outcomes.

The last of the ARMADILLO study participants finished their study period and follow-up period by August 2018. After minor modifications, the full ARMADILLO architecture, consisting of all domains and their subdomain messages, was linked via an overarching domain-selection menu message and made available to participants from all arms for 2 months. The system was taken offline in December 2018 and remains offline while primary and secondary analyses from the trial are being conducted.

The ARMADILLO study obtained ethics review and approval from the World Health Organization’s Research Ethics Review Committee (A65892b) and the University of Nairobi/Kenyatta National Hospital (P274/05/2017).

### Recruitment

Participants were identified via a household enumeration of eligible youth, which took place October 2017. In this enumeration, the research team used an official record of households (developed in preparation of Kenya’s 2019 national census) to map all households in the study area. Trained data collectors recruited from the study area then visited every household (a total of 2132) to identify eligible youth. Household members were deemed eligible if they were between the ages of 18 and 24 years, literate, had their own mobile phone (meaning it was primarily in their possession and they controlled when and with whom they shared access) and reported regular use, had a mobile phone with them at the time of recruitment, and reported current use of text messages.

When RCT recruitment began in February 2018, one eligible youth per household was preselected randomly for recruitment; if they opted not to participate, no one else in the household was eligible. Enrollment of participants was rolling and took place in three waves over seven weeks.

### Data Collection

All surveys were completed on a mobile phone via digital form (ODK Collect); surveys were primarily administered by data collectors, although participants filled in the digital forms themselves for certain sensitive questions. Twenty-one individuals from the study site community were hired to serve as data collectors for the RCT. An almost-equal number of male and female data collectors were selected to ensure that all participants would be recruited, consented, and enrolled by someone of the same sex. Most data collectors had completed at least some secondary education.

Before participant recruitment, data collectors underwent a three-day training that covered an overview of the study and its purpose, the process for ethically recruiting and consenting individuals, and how to collect data (for participant surveys) via a digital form on mobile phones. Given the taboo nature of an SRH-related study conducted in a conservative community, the training included a special focus on making sure that young participants would feel comfortable speaking with data collectors.

### Study Arms Description

After being randomized, participants were intended to automatically enter into one of the three arms the following day: they would receive either their first domain menu (arm 1), domain contact (arm 2) message, or no message (arm 3), marking the start of their intervention period.

**Figure 1:**
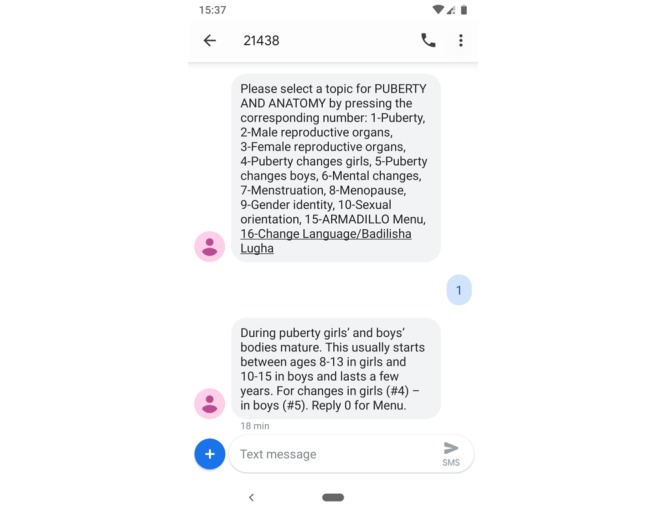
Example interaction with the ARMADILLO (Adolescent/Youth Reproductive Mobile Access and Delivery Initiative for Love and Life Outcomes) puberty and anatomy domain (seen by arm 1 participants).

Arm 1 provided the ARMADILLO intervention: SMS text messaging content around seven youth-identified SRH domains: puberty/anatomy, pregnancy, relationships, sex, contraception, HIV, and gender-based violence. Arm 1 participants received one SMS text message pushed to their phones every week providing them with a new, unlocked domain menu; at their convenience, they could request further information on any of 5 to 12 numbered subdomains, which then provided them with two to three SMS text messages of validated health information, developed by youth. An SMS quiz was pushed to arm 1 participants’ phones at the end of the week to maintain engagement. Any participant who responded received a phone credit equivalent to US $0.50. An example interaction with a domain message, user reply, and subdomain message can be seen in Figure 1.

Arm 2 matched the system-initiated contacts with participants of arm 1 without providing them access to the ARMADILLO content itself. The intended purpose of arm 2 was to assess whether changes in SRH outcomes were due to exposure to messaging content or the “contact” of the digital intervention itself; that is, would a young person, encouraged by SMS text message to go learn about a given SRH topic, be inspired to seek out information on their own (eg, by talking to friends and family, or looking up information online)? Practically, this meant that arm 2 participants received two pushed messages per week: one alerting them to an SRH domain on which to seek information and an SMS text message quiz at the end of the week. Any response to the SMS quiz received a phone credit equivalent to US $0.50. For both arms 1 and 2, in a reflection of the demographics of the study area, messages were available in both English and colloquial or “street” Coastal Swahili.

Finally, arm 3 was a control arm. Arm 3 participants emulated standard access to SRH information and thus received no messages from this study. The ARMADILLO system was stored on RapidPro (an open-source communication platform), hosted by the technology partner, Ona.

## Results

### Implementation Challenges

Two seemingly minor methodological decisions had significant implications for the overall success of the trial’s enrollment and intervention implementation. We describe these below.

#### Challenge 1: A Hands-Off Recruitment Process

During the enrollment period, data collectors were instructed to identify the randomly selected youth from each household based on this young person’s age, sex, and education level (intentionally, no further identifying information had been collected from the young people during the enumeration process). Data collectors confirmed the youth’s identity by collecting these demographic details again. They determined eligibility by asking if the youth owned a phone, asking to see the phone, and collecting the phone number. There was no additional check to verify phone ownership or confirm the phone number provided was in service.

If the young person was confirmed to be eligible and expressed an interest in participating, data collectors consented the youth and began the baseline survey. As part of the consent form (read aloud by the data collector to the youth), all three arms of the ARMADILLO study were described. These were the only instructions on any of the three arms that participants received, an intentionally hands-off approach to emulate as much of a real-world environment for the intervention as possible. As a result, any participant unfamiliar with the intervention’s format (or unclear that they could expect to *receive* any intervention) struggled.

#### Challenge 2: Design Flaw in Language Selection Menu

The ARMADILLO system was built for two languages, Swahili and English, but there was no clear preference in the study area for which language could serve as a default. As such, the day after enrollment we decided that if a participant was randomized into either arm 1 or 2, they would receive a single SMS text message asking them to indicate in which language they wished to receive messages. A response to this initial language selection SMS text message triggered their first domain menu or domain contact and the start of their seven-week study period. However, a critical by-product of this decision, and a design flaw, was that if a participant did not respond to this message, they were left in a study timeline “stasis.” Their seven-week intervention period would not trigger until they responded to the initial SMS text message; as such, they would not time out of the study (and therefore be able to participate in endline data collection) because they had never timed in.

**Figure 2:**
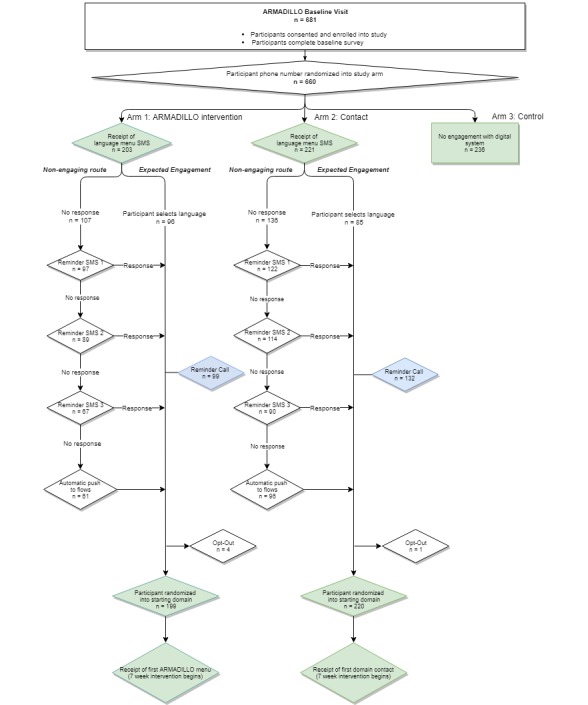
Progression of participants: how entry into the randomized controlled trial was planned (expected engagement) versus additional steps taken (nonengaging route) for each arm.

As a result, three weeks after the first study participants were enrolled, passive monitoring of participants’ progression through the system revealed a number of arm 1 and arm 2 participants who were trapped at language selection because they had not responded to the initial SMS text message from the study. At this point (as seen in Figure 2), 203 participants had been randomized to arm 1, 221 to arm 2, and 236 to arm 3. Among the 424 participants in arms 1 and 2, only 181 (42.6%) had successfully selected a language and initiated their seven-week intervention period; an estimated 243 still had not proceeded past the entry language menu. Arm 1 had fewer participants stopped at this language menu than arm 2 (107 arm 1 participants versus 136 arm 2 participants); however, over half the participants in each arm were “stuck.”

To resolve this, we took a series of successive steps to nudge participants into the system, before eventually integrating a nonresponse mechanism (which should have been done initially). With this mechanism, anyone at the initial language menu now automatically flowed to a Swahili-language domain message (and therefore the seven-week intervention period) after one day of inactivity. Figure 2 describes how the RCT was planned (the green pathway) versus the additional steps the study team had to take to move nonengaging participants into their selected arm.

### Reasons for Nonengagement

As seen in Figure 2, nudges started with a few low-interference reminder SMS text messages, which were successful in prompting several participants in each arm to select a language and begin their intervention period, implying that these participants had just required a reminder. Additionally, approximately five weeks after the first participants had been enrolled, the study team called a cross-section of nonengaging participants to encourage them to respond to the language message. During these calls, the study team also learned reasons, unrelated to the language menu option, that participants had not yet responded to the initial message.

When the research team called nonengaging participants, 99 participants in arm 1 remained stuck at the language menu, along with 132 participants in arm 2. The research team called all 231 of these nonengaging participants over two days. The team was successful in reaching 136 participants (59 in arm 1; 77 in arm 2), and we were able to learn their reasons for nonengagement. Among this selected subset of reachable nonengagers, Table 1 lists key reason for nonengagement.

**Table 1:** Reported reasons for not responding to the ARMADILLO language menu, according to the nonengaging participants in arms 1 and 2 who responded to phone calls from the ARMADILLO team.

Reported reason for nonengagement		Arm 1 (n=59), n (%)	Arm 2 (n=77), n (%)	Total (n=136), n (%)
**Eligibility challenges**		15 (25)	17 (22)	32 (24)
	Person who answered phone was not participant and did not share phone with participant (participant gave someone else’s phone number)	7 (12)	12 (16)	19 (14)
	Person who answered phone was not participant but did share phone with participant	8 (14)	5 (7)	13 (10)
Did not recognize the system (assumed scam/spam)		8 (14)	22 (29)	30 (22)
**Confusion over how to engage with the system**		17 (29)	10 (13)	27 (20)
	General confusion (nonspecified)	8 (14)	3 (4)	11 (8)
	Did not know how to progress/was not sure it was free	4 (7)	3 (4)	7 (5)
	Thought the system was supposed to call them	1 (2)	4 (5)	5 (4)
	Other (thought messages were time-sensitive; did not know to expect message, thought system was “pushed”)	4 (7)	1 (1)	5 (4)
Apathetic about engaging		6 (10)	5 (6)	11 (8)
**Technical challenges**		9 (15)	15 (19)	24 (18)
	Reported not having received messages	3 (5)	6 (8)	9 (7)
	Hardware issues: phone was lost or broken	1 (2)	4 (5)	5 (4)
	Telco issues: line no longer in service, incomplete number, noneligible mobile network operator	3 (5)	3 (4)	6 (4)
	Other (had submitted invalid responses, had multiple phones)	2 (3)	2 (3)	4 (3)
Other reasons		4 (7)	8 (10)	12 (9)

Several nonengagers were found to have violated eligibility criteria, specifically phone ownership. First, 19 phone numbers did not belong to the person who had been recruited to participate (confirmed by the phone owner being outside the 18-24 age range or from outside the study area, and having no recollection of being interviewed). Discussions with the true phone owners indicated that a young study participant may have—out of fear, distrust, or mischievous spirit—opted not to give their own phone number but rather that of a friend, relative, or acquaintance. In other cases, participants may have provided a wrong number, or a data collector may have entered a wrong number. In either case, the phone owners did not recognize the messages from ARMADILLO and did not respond. Phone owners were able to opt out if they wished. However, the individual participants who had provided the numbers were not unenrolled and completed an endline assessment.

An additional 13 calls reached persons who shared a phone with the study participant; for example, one recruited participant was the full-time owner and operator of a phone, but only when his brother was away at university. When the brother returned during the study period, the participant forfeited the phone (purchased by the brother). The brother (and the other nonparticipants in this group) had not recognized the messages arriving to the phone and had not responded. ARMADILLO eligibility criteria had specified phone ownership; therefore, the fluid phone-sharing arrangements meant that these participants violated eligibility criteria as well.

Among eligible participants, the single largest reason for nonengagement was that participants had not recognized the introductory SMS text message as being from the study. Thirty participants indicated they had assumed the introductory message was the start of a scam or that they were being spammed by a third party. An additional 27 of the nonengagers reached indicated being confused by the system. Specific reasons included not being sure how to progress through ARMADILLO, uncertainty that the system was free, thinking that the system was supposed to call them, an assumption that messages had to be responded to within a certain period, and believing ARMADILLO was a push system. An additional 11 expressed some level of apathy with the system, telling the research team that they had been too lazy, too busy, or not interested enough to reply.

Finally, the research team found 24 of the nonengagers had experienced some sort of technical issue. These ranged from numbers being out of service or not on a participating network, participants losing their phones or having other phone issues, and participants reporting either not having received the message or having their responses rejected by the system.

Some participants eventually moved into the system following the call. The remainder, including those who could not be reached by phone, automatically flowed into their first domain shortly thereafter, following the system modification.

## Discussion

### Principal Findings

This study describes some of the pragmatic implementation challenges that can arise while implementing a rigorous, multiarm RCT assessing the efficacy of a digital health intervention. Although the quantity and general quality of evidence appear to be increasing in recent years [21,22], RCTs in particular can pose challenges for the digital health field; for example, blinding participants to the intervention they receive is extremely difficult [20]. In addition to being costly, RCTs traditionally also have lengthy recruitment, enrollment, and study periods [23], and the interventions they test—predefined in the trial protocol—remain static for the duration of the study [20]. This can be problematic in a field where innovation and invention advance the field quickly in a short period of time. Finally, as the success of digital interventions can depend as much on contextual factors as on the intervention itself, the appropriateness of RCTs alone to contribute evidence has been debated, with calls evaluations to include robust qualitative components [20,23].

We attempted to account for several of these challenges in the design of the ARMADILLO study. Our RCT had relatively short intervention and follow-up periods, a nod to our transient young population. The development of the study was recognized to be a multiyear process; therefore, we selected SMS text messaging as a delivery channel that, although not at the vanguard of digital health innovation, was and would remain a reliable and universal channel of communication for anyone with a mobile phone. Finally, before developing the RCT, we conducted a robust qualitative phase, which not only vetted the ARMADILLO content but also sought to understand the sociocultural and technological context in which the RCT would be implemented.

However, even while accounting for common challenges to digital health RCTs, two decisions had consequences that threatened the rigor of the planned RCT. One led to data collectors being as hands-off as possible in confirming participant identity and describing the intervention. A second decision introduced an improvised language selection menu to ensure that participants could access messages in their language of choice.

Confronted with dozens of participants stalling at the language menu, we faced a question: why nudge and then push participants into the system at all? Nonengagement with the language menu could be factored into the analysis, for example, by comparing findings using intent-to-treat analysis with those of a per-protocol analysis. The reality was not so simple: the language menu was a last-minute add-on for a study site with two equally used languages; it was separate from the intervention being evaluated (seven weeks of SRH content delivered via text message). As such, nudging or moving participants past the language menu and into their study arm was deemed appropriate. Once participants had flowed into their timed intervention period, we could monitor participants’ levels of engagement (or lack thereof); these will be factored into upcoming analyses of the trial results.

Although unplanned and time-intensive, a serendipitous result of calling participants was that the study team was able to communicate directly with nonengaging participants to find out why they had not yet responded. Most reasons had nothing to do with the language menu but rather the intervention or study. However, if the nonresponse mechanism had been built into the language menu initially (as it should have been) so that nonresponding participants flowed directly from language menu to their first domain, we would not have captured the views of these nonengagers.

Broadly, the ARMADILLO study’s implementation challenges, which arose during the study’s enrollment and initial data collection period, provide several lessons relevant for both research and implementation of client-side digital health interventions.

### Research Challenges and Lessons Learned

Lessons learned from the first implementation misstep—the hands-off manner in which participants were recruited and enrolled into the study—can assist future researchers to more carefully design their studies and recruitment procedures, as described subsequently.

#### Develop Meticulous Phone Data Collection Protocols to Reduce the Possibility of Wrong Numbers

In our calls to nonengagers, we connected with several people who were from the study area but who were not our recruited participants. We have no real way of knowing why we ended up with these wrong numbers. Perhaps it was overenthusiasm: participants who did not quite meet our eligibility criteria but still wanted to participate may have borrowed the phone or phone number of a friend or family member. Conversely, it might have been underenthusiasm; we may have overestimated young people’s comfort with participating in a study that would involve SRH messages arriving to their phones.

Data collector trainings establish and drill procedures for recruiting, consenting, and enrolling participants. For digital health interventions, these procedures must also include multiple steps for cross-checking that phone-related eligibility criteria are met, and that the correct phone number is collected. Using ARMADILLO’s phone-related eligibility criteria as an example, simple measures can reduce phone-related recruitment error:

1. asking to see the participant’s phone;
2. repeating the phone number back to the participant;
3. calling the participant’s phone and checking the phone to confirm receipt; and
4. probing to ascertain whether the participant meets ownership criteria as defined by the study (eg, Who buys air time? Who purchased the subscriber identification module (SIM) or phone? Who else can use the phone, and who decides this?)

#### Train Participants in Efficacy Assessments on the Digital Intervention (Even If It Is Modeled Off Similar Services)

ARMADILLO’s on-demand querying of information (arm 1) uses a number-based menu that makes it virtually identical in format to M-PESA, a mobile money service used by more than 18 million people in Kenya [24]. The assumption was that the ping-pong format of user-system interaction would feel familiar to users, and minimal explanation would be necessary. We were also concerned about overly training participants in arm 1 only for them to be confused, disappointed, or less willing to engage if they were to be randomized into one of the other two arms.

Unfortunately, for a minority of participants in arms 1 and 2, the lack of detailed explanations resulted in confusion as to how to engage with the system. Additionally, participants in both arms did not make the connection between the study and the study’s SMS text message, even after receiving this SMS text message the day after they were interviewed.

It is tempting, during an evaluation of client-side digital health interventions, to adopt a hands-off approach with users; a user’s ability or inability to successfully engage with a system is data in and of itself. This approach can be appropriate for evaluations of service rollout (coverage assessments) or studies of usability or acceptability [1]. However, this randomized trial was an efficacy assessment meant to assess whether the digital intervention affected health outcomes under an ideal research setting. Therefore, with a focus on health rather than usability outcomes, participants must be fully trained to be able to use the system as intended. Data collectors should be instructed to be explicit about the service at the point of recruitment and walk all participants through the following, in detail:

1. How the system works: describing all arms in detail, what cost (if any) is incurred for participants, when and how often they can engage.
2. How to recognize the system: what number or short code does the system use, when can they expect to receive messages (for any pushed interactions).
3. How to use the system: showing example messages on a phone, letting the participant try querying the system on the data collector’s phone.

#### Client-Side Digital Health Interventions Have Analog Discontinuation Challenges

For a study focusing on a young and geographically mobile population, a participant’s phone was not only an essential part of the intervention but also an important tool for locating the participants and scheduling endline and follow-up interviews. That said, the young participant’s phone itself can become a source of discontinuation. Youth participants lost possession of the phone subscribed to the ARMADILLO system because it was lost or stolen, they upgraded to a new phone, the phone broke (temporarily or permanently), they switched SIMs or providers, or they loaned the phone to a friend or relative for short or long periods of time. Common phone-related discontinuation challenges should be considered with other sources of discontinuation when calculating sample size to ensure that an otherwise robust study does not become underpowered because several participants lose their phone.

### Service Rollout Challenges and Lessons Learned

Although ARMADILLO was an RCT and not a full-scale digital health campaign, lessons learned from the calls to nonengagers (made as a result of the second implementation misstep) can contribute to the successful development and rollout of both categories of digital health communication services.

#### Determine Whether an Intervention Requires Phone Ownership or Phone Access

When developing targeted client communication digital health interventions (for example, SMS text messages to expectant mothers throughout their pregnancies; alerting clients about health tests results) [3], especially interventions around sensitive issues, such as SRH including HIV, it is critical to understand what comprises phone ownership in a given setting.

Outreach to ARMADILLO study users found that phone ownership was a fluid concept; a phone might belong to a user for a certain period during the day, during a certain time of year, or until someone gets an upgrade and passes down their old phone. There is a general need for data on the demographics and practices of phone ownership and phone sharing.

In Kenya, data from a 2009 nationally representative survey of over 30,000 individuals aged 16 years and older showed that although 85% of individuals indicated that they had used a mobile phone, only 44% owned their mobile phone. Phone sharers were predominantly female (65%), and lower levels of phone ownership were observed among the youngest respondents [25]. More recent regional data suggests that women in sub-Saharan Africa are 14% less likely than men to own a mobile phone (defined as having sole or main use of a SIM card or mobile phone which does not require a SIM), and women are 34% less likely than men to use mobile internet [26].

Client-side digital health interventions provide an important mechanism for conveying health information to hard-to-reach populations. However, digital health implementers should take care to consider whether their intervention requires mobile phone ownership (and what that means) or mobile phone access for effective, acceptable, equitable, and safe engagement with users. Digital health interventions will reach their intended populations only when implementers understand the realities of (1) how age, gender, income, or urban-rural status influence likelihood of phone ownership and (2) how phones are shared within households.

#### Digital Health Campaigns Must Establish a Credible Presence in a Competitive and Busy Digital Space

Similar to preparation for a health communication campaign, the ARMADILLO study team conducted extensive outreach with county-level Ministry of Health officials as well as community leaders to ensure that communities were sensitized to the coming research. However, given the design of this efficacy assessment involved a control group receiving no intervention, special care was taken to avoid contamination across groups of participants in the study area by not advertising the ARMADILLO system within the community itself—not a strategy to be recommended outside a research setting.

A downside to staying quiet about the service was that ARMADILLO did not automatically have the trust or recognition of its participants. ARMADILLO’s formative stage found that young people (and, importantly, their parents or caregivers) were enthusiastic about a phone-based health campaign, so long as they knew it was coming from a credible, trustworthy source [27]. That the single largest reason for nonengagement in this study was not recognizing or trusting the sender reinforces those findings.

An additional reason for distrust was likely how the ARMADILLO registered on participants’ phones, a weekly SMS text message from a numeric short code. Other large-scale pushed-SMS text message campaigns use customized names for easy recognition (eg, SMS text message coming from “ARMADILLO”). However, this was not possible given ARMADILLO’s ping-pong format, in which participants were expected to interact with the system. At the same time, public awareness and news coverage of mobile-based financial scams is increasing [28], with Kenyans being advised to be on guard against social engineering by scammers in an attempt to gain personal and financial information [29]. Therefore, incoming messages from a numeric short code may have been viewed with added skepticism.

In implementing client-side digital health communication campaigns, the importance establishing its trustworthiness within the community (both intended users and the community at large) cannot be overemphasized.

#### Interest in a Service Can Be Sporadic and Fleeting

Finally, 11 of the nonengaging participants (8% of the total number of nonengagers reached by phone) saw the messages but then were either too lazy, too busy, or forgot entirely to respond. These participants provide an important reminder that—however exciting a digital system is—intended users may not wait by their phones for messages or opportunities to engage.

Purely on-demand interventions rely on user initiative and therefore user interest for accessing information. However, just as all mobile phone users may forget or get too busy to engage in personal messaging, even pushed message campaigns, whether providing targeted or untargeted client communication, would do well to remember that users’ interest and bandwidth to engage will wax and wane over the course of a campaign.

### Conclusions

Digital health interventions are lauded for their potential to overcome health client, provider, and system challenges that hinder the coverage or effect of existing health interventions. However, the digital health field is still in its adolescence—and enthusiasm often outpaces evidence. Most recently, a Lancet editorial cautioned against “digital exceptionalism” and highlighted the risk to patients and the health system if we fail to robustly evaluate digital health interventions [30].

The ARMADILLO study was developed with a sole focus on robust evaluation and despite the challenges previously described, preliminary data review has indicated that enough participants received necessary parts of the intervention to be able to power the planned primary and secondary analyses. However, even the process of implementing this multiarm RCT has eliminated certain illusions of digital exceptionalism. Research on digital health interventions faces the same implementation challenges as other research on nondigital health interventions: difficulty reaching the target population, trouble following-up with participants, and overcoming reluctance to engage. If these challenges are not adequately prepared for in future research, there will be adverse implications on the availability and quality of evidence in a field where evidence is sorely needed.

## Abbreviations

ARMADILLO: Adolescent/Youth Reproductive Mobile Access and Delivery Initiative for Love and Life Outcomes
RCT: randomized controlled trial
SIM: subscriber identification module
SMS: short message service
SRH: sexual and reproductive health
